# “Of Mice and Men”: Arginine Metabolism in Macrophages

**DOI:** 10.3389/fimmu.2014.00479

**Published:** 2014-10-07

**Authors:** Anita C. Thomas, Joshua T. Mattila

**Affiliations:** ^1^Bristol Heart Institute, School of Clinical Sciences, University of Bristol, Bristol, UK; ^2^Department of Microbiology and Molecular Genetics, University of Pittsburgh, Pittsburgh, PA, USA

**Keywords:** arginase, human macrophage, macrophage polarization, nitric oxide synthase, macrophage activation

## Introduction

Macrophages are involved in inflammation from induction to resolution. Polarization of macrophages along the M1 (classical) or M2 (alternative) axis occurs during inflammation and can be at least partly categorized by the route of arginine metabolism within the macrophage, balancing the activities of the arginase and nitric oxide synthase (NOS) enzyme families (1, 2). Arginase activity is associated with tissue repair responses (via ornithine production and pro-proliferative effects). In contrast, NOS2 generates nitric oxide (NO) species with anti-proliferative effects that is necessary for protection against pathogens and aberrant cells (2, 3). Other NOS enzymes produce NO that acts in the regulation of smooth muscle tone and other cellular processes (4). Macrophages preferentially expressing the arginase or NOS2 pathways enzymes also influence T-cell activation, proliferation, signaling, and apoptosis in different ways (1).

While arginase and NOS enzymes can be used to ascertain the pathway of macrophage activation in rodents, there has been debate as to whether they are present in macrophages from humans and other mammals. The arginase and NOS enzymes are extensively conserved, and the NOS forms found in mammals are similar to those in cnidarians, mollusks, and other chordates (5, 6). These arginine-metabolizing enzymes are present in some human leukocytes, and there is evidence that they are also present in macrophages from other vertebrates, including chickens, rabbits, cows, and primates (7–12). However, comparisons of tissue macrophages of different species are lacking, which limits our understanding (13). Many studies in humans have principally focused on blood monocytes, leading some researchers to question the suitability of rodents as model of macrophage activation, as there is not always a direct correlation with human cells. Was Robert Koch correct when he said “Gentlemen, never forget that mice are not humans,” or can the differing results between species be explained, in part, by differences in the types of monocyte or macrophage studied? Our purpose here is to examine this question.

## Arginine Metabolism in Mammalian Cells

Many mammalian cells, including neutrophils, granulocytes, erythrocytes, hepatocytes, cardiac myocytes, dendritic cells, myeloid-derived suppressor cells, foam cells, natural killer cells, endothelial cells, and smooth muscle cells, have arginase (12, 14–16) or NOS activity (8, 17–19), albeit to different degrees. Macrophages are the primary circulating cells that can express either of these enzymes, depending on the inflammatory circumstance. Experiments that detect NO, ornithine, or urea production (via NOS2 or arginase) have most often been performed on rodent macrophages. Macrophages from some mouse strains (e.g., the M1-biased C57BL/6 strain) can be stimulated by lipopolysaccharide (LPS) to produce considerable quantities of NO. Macrophages from others strains (e.g., M2-skewed BALB/c mice) produce much less NO (20) and produce more ornithine instead. Some researchers did not detect any NO production in macrophages from humans, pigs, and rabbits (8, 11, 14, 21–23), but others (including ourselves) have observed NOS or arginase activity in macrophages from rabbits, humans, and other primates (4, 7, 10, 12, 17, 24–26).

## Why is there Controversy?

One main difference between the studies from laboratories is that some use monocyte-derived macrophages (MDM), while others study tissue macrophages directly. A number of groups have detected NOS or arginase activity in human monocytes or macrophages (3, 27–29); but others have not. Why is this so? Part of the explanation lies in the fact that *in vitro*-derived macrophages can generate different responses from macrophages obtained *in vivo* as discussed below (and shown in Table 1). Another explanation is that many groups use the identification of enzyme protein rather than detection of enzyme activity as evidence of enzyme expression. Failure to detect the presence of a protein is not definitive evidence for absence of expression (especially when considering potentially different detection thresholds of antibodies or the high *V*_max_ of arginase, i.e., very little enzyme is required for ornithine production).

**Table 1:** **The presence of arginine-metabolizing enzymes in human monocytes and macrophages varies with cell source, treatment and health status/stress level of the individual**.

Cell origin	Cell	Treatment	NOS test	ARG test	Result	Reference
Blood monocytes	Monocyte, mono-mac	0, 2, 3, or 5d culture	RNA, citrulline, FC	RNA, urea	NOS, ARG1 and ARG2 levels vary between monocyte subpopulations	(27)
Blood monocytes	Monocyte, mono-mac	0, 3, or 7d culture or 7d M-CSF, 0.75d IFNγ/LPS, or IL4		Gene array	No difference (≤2-fold cut-off, therefore genes with smaller differences discounted)	(30)
Blood monocytes (filaria-infected)	Monocyte	1d culture	RNA	RNA	↑ARG1, ↓NOS2	(28)
Blood monocytes (burns victims)	Monocyte	2d culture		Urea	↑ARG1	(29)
Blood monocytes	Monocyte	2d microfilaria, M-CSF, IL4, or IFNγ/LPS	RNA	RNA	Most donors had low but detectable NOS2 and ARG1 RNA expression which did not change with any treatment.	(31)
Blood monocytes	Mono-mac	3d IFNγ and/or IL4 (No M-CSF)		RNA	↓ARG1, but detectable in all conditions	(32)
Monocyte/macrophage cell line (U937)	Mono-mac	?d LPS and/or IFNγ	Transcription run-on assay		No induction of NOS2 gene transcription (for that particular region of the promoter region)	(33)
Monocyte/macrophage cell line (U937)	Mono-mac	1d selenomethionine and 1d LPS and/or IFNγ	Griess, RNA Western		Selenomethionine ↓LPS-induced NOS2 expression (RNA and protein) and nitrite production	(34)
Blood monocytes, peritoneal macrophages	Mono-mac, macrophage	?d culture, 2d LPS, IFNγ, or TNFα/GM-CSF	Griess, amino acid HPLC		No nitrite, ornithine, citrulline production, no arginine consumption	(22)
Blood monocytes, peritoneal macrophages	Monocyte, mono-mac	0d or 3d LPS or cytokine	RNA, IB, ICC, biopterin, citrulline, Griess		NOS2 mRNA and protein present in monocytes, ↑peritoneal macrophages (↑with LPS). Both cell types produce neopterin, nitrite/nitrate and citrulline (low levels)	(35)
Blood monocytes (MS sufferers)	Macrophage	6d GM-CSF 0.75d IL4, IFNγ, LPS, or TNFα	RNA, Griess	RNA, WB, urea	ARG1 and NOS2 mRNA and nitrite production in MS and controls, ↑with M1 or M2 cytokine challenge. ARG1 protein and urea production present in controls, ↑in MS	(36)
Blood monocytes	Macrophage	8d M-CSF, 5d oxLDL		RNA	No change in ARG1 levels	(10)
Blood monocytes	Macrophage	10d M-CSF, 1d IL4, or IL10		Urea, WB arginine	No ARG1 after induction by IL4 or IL10	(14)
Blood monocytes	Macrophage	14d IFNγ/LPS	Griess		No nitrite production	(37)
Alveolar macrophages (volunteers)	Macrophage	IFNγ	Griess, citrulline		No NO production, no effect of NOS inhibitor	(21)
Alveolar macrophages	Macrophage	?d (short), 0.8d IL4, or forskolin (i.e., ↑cAMP)		Urea	Untreated macrophages have ARG activity similar to unstimulated RAW cells. ↑ARG with IL4/forskolin but not IL4 alone	(38)
Alveolar macrophages (cancer suffers, volunteers)	Macrophage	0.75d IFNγ/LPS or IL-10	RNA, WB	RNA	↑ARG with IL10 stimulated cells, ↑NOS2 with IFNγ/LPS stimulated cells	(39)
Alveolar macrophages (TB patients, volunteers)	Macrophage	None	IHC, WB, RNA, diaphorase		45–49% of cells from TB patients have NOS2. Smoking controls had some NOS2-positive macrophages, non-smoking controls have few NOS2-positive cells	(24)
Alveolar macrophages (TB patients)	Macrophage	None	IHC		Macrophages in TB granulomas stain for NOS1, NOS2 and nitrotyrosine (i.e., active)	(26)
Alveolar macrophages (TB patients)	Macrophage	None	IHC	IHC	ARG1 in macrophages in TB granulomas, few have Arg2. Some macrophages on outer margins have both NOS2 and ARG1, some near center have NOS2, NOS3 and ARG1	(12)
Atherosclerotic plaque macrophages	Macrophage	None	ISH, IHC		NOS2 in macrophages and smooth muscle cells, co-localized with oxidized lipoproteins and peroxynitrite (i.e., NOS is active)	(7)
Atherosclerotic plaque macrophages	Macrophage	None	IHC, WB		Fatty streaks: no NOS2. Advanced plaques: NOS2 present in macrophages near necrotic core, associated with ceroid accumulation and nitrotyrosine (i.e., active)	(25)
Atherosclerotic plaque foamy macrophages	Macrophage	None		IHC	↑ARG1 in macrophages in superficial layers, ↓ARG1 in macrophages surrounding lipid core	(10)
Atherosclerotic plaque	Macrophage	None	IHC, ISH		NOS2 and nitrotyrosine localized to smooth muscle cells, macrophages and foam cells (i.e., active)	(17)
Oral macrophages	Macrophage	None	IHC, nitrate		NOS2 present in macrophages from gingivitis samples	(40)
Placental macrophages	Macrophage	None		FC	Some M2 macrophages have ARG1	(16)
Skin macrophages (wound)	Macrophage	None	IHC, HPLC	IHC, WB, ELISA, HPLC	NOS2 present in macrophages, some have ARG2, but none have ARG1. Controls: no ARG2	(41)
Tumor-associated macrophages	Macrophage	None	IHC		NOS2 present in some macrophages (bladder)	(42)

*While changes in RNA expression of arginine-metabolizing enzymes have been used to identify macrophage activation states, protein changes [such as western blotting (WB) or immunohistochemistry (IHC)] are also useful. Nitric oxide synthase (NOS) activity can be assessed directly [e.g., production of citrulline or NO (e.g., Griess assay)] or by the presence of markers of NO production (such as peroxynitrite, nitrotyrosine or ceroid, a complex of oxidized lipids and proteins). Arginase (ARG) activity can be measured as urea or ornithine production (e.g., urea assays, amino acid HPLC)*.

*d, number of days; ?d, unspecified number of days; FC, flow cytometry; M-CSF, macrophage colony-stimulating factor; IFNγ, interferon-γ; LPS, lipopolysaccharide; IL, interleukin; TNFα, tumor necrosis factor-α; GM-CSF, granulocyte-macrophage colony-stimulating factor; IB, immunoblot; ICC, immunocytochemistry; MS, multiple sclerosis; oxLDL, oxidized low density lipoprotein; TB, tuberculosis; ISH, *in situ* hybridization; Griess, Griess assay for nitrite/nitrate production*.

*It should be noted that NO production below the detection levels of this relatively insensitive assay may still have functional effects (43)*.

### Macrophages produced *in vitro*

Macrophages have been produced *in vitro* in a number of ways. Cells from bone marrow have been isolated and “differentiated” in culture medium containing high levels of cytokines (such as colony stimulating factors, CSFs) to produce bone marrow-derived macrophages (BMDM) (13, 23, 44–46). Macrophages have also been produced by isolating and culturing monocytes from blood, to produce MDM (10, 13, 22, 30, 37, 47, 48). Production of these *in vitro*-derived macrophages is cheap, simple, and reproducible, but they may not be a full representative of tissue macrophages, as the preparation and culture procedures may not be sufficient to induce cell activation (4). The differences between tissue macrophages and *in vitro*-derived macrophages are at least partly dependent on cell source, time in culture, and the degree of manipulation in culture. Each research group will use different types and sources of culture media and sera, which vary greatly in the concentrations of factors that influence NOS2 or arginase expression, such as transforming growth factor β (TGFβ) (4, 20, 49). Another confounding issue is that circulating monocytes and tissue macrophages arise from different stem cell populations (50), although some macrophages found at sites of infection or inflammation may derive from infiltrating monocytes (51). Together, these factors may account for many of the differences observed in NO and urea production in these macrophages (8, 20).

Monocyte-derived macrophages or BMDM from different strains of mice can differ in their response to interferon-γ (IFNγ), LPS, and tumor necrosis factor-α (TNFα) (4, 8), and differences in the rodent background can result in differences in macrophage gene expression (13, 20, 49). Human *in vitro*-derived macrophages also show variability in their responses to LPS (4, 22, 46). It may be that the same stimulus is able to generate quite different responses in genetically diverse individuals, as it does between mouse strains (38, 49, 52). In general, human macrophages are not as responsive to LPS as mouse macrophages, possibly because of the lower environmental exposure of humans to LPS. It is also possible that human monocytes may be more effectively stimulated to become M1-activated macrophages by cytokines other than IFNγ and LPS/TNFα (e.g., IFNα) (4, 18, 43). Human macrophages take longer time to respond to the stimulatory factors *in vitro* than mouse macrophages, and some experiments using human MDM may have ended before a response was detected (48). There are other indications that the timing and length of the exposure of the cells to varying cytokines *in vitro* are important. For example, when M1-polarizing cytokines were removed from the culture medium, NOS2 levels in mouse BMDM were reduced and NO production (measured as nitrite) ceased (45). In addition, whichever arginase or NOS enzyme was induced earliest, the alternative enzyme decreased in expression and activity, unless arginine was present in excess (15, 45, 53). Macrophages require the local environment to continuously give appropriate activation cues. Changes in environmental cues can stimulate macrophage populations *in vitro* to express varying percentages of M1 or M2 dominant activity (54). When activation cues are reduced or removed, macrophages may become deactivated (e.g., M2c) or indeterminate (e.g., have features of M1 and M2).

### Macrophages obtained *in vivo*

Macrophages can be identified in whole tissues and organs or isolated in large numbers from *in vivo* sources such as the peritoneum or granulomas, and either examined immediately or used *ex vivo*. Macrophages obtained *in vivo* or made from monocytes can respond differently to the same stimulus (35, 47). In one study, monocytes and tissue macrophages were obtained from patients with an inflammatory disease (either rheumatoid or psoriatic arthritis). Compared with tissue macrophages, the MDM had a blunted response to the M2 cytokines interleukin-4 (IL-4) and IL-13, at least partly due to a reduction in some of the receptor elements for these cytokines (47). These results suggest that the response of the macrophages to M2 cytokines may be source specific, but it is possible that these cytokines alone were not sufficient to fully stimulate the MDM (38). Several lines of evidence suggest that macrophages *in vivo* express functional NOS2. Blood monocytes and peritoneal macrophages obtained from women during laparoscopic procedures contained NOS2 mRNA and protein. The macrophages had higher NOS levels than the monocytes, and this could be increased by treatment with LPS. The monocytes and macrophages also produced neopterin, nitrite/nitrate, and citrulline (suggesting that the enzyme was active). Although the production of NO from these macrophages was low, it would probably have been sufficient to cause functional changes (35).

Macrophages can also be obtained from alveolar aspirates, skin, and the placenta (10, 16, 21, 38, 39, 55, 56). For example, sponges placed subcutaneously into mice, rats, or rabbits attract large numbers of macrophages. The sponges can be removed from the animal and the macrophages were isolated and purified (10, 55, 56). It is a little more difficult to obtain and purify macrophages from other tissues, such as atherosclerotic vessels (44), but intact biopsy, surgical, or cadaveric specimens can also be investigated. It should be noted that resident macrophages from different tissues observed at different times (and different health states) may not necessarily have identical properties (51, 57).

In order to perform their full range of functions, macrophage populations exhibit “plasticity” of phenotype (52, 58), regardless of whether they are found *in vivo* or derived *in vitro*. As macrophages adapt or change their functions, they can simultaneously express markers of M1 and M2 activation, including NOS2 and arginase-1 (12, 59, 60). For example, tissue macrophages (and MDM) from *Mycobacterium tuberculosis*-infected cynomolgus macaques have been observed to co-express functional NOS and arginase enzymes (12). We suggest that *macrophages display a spectrum of activation phenotypes, and it is the relative (and not absolute) proportion of M1 or M2 markers that we can use as a ‘handle’ to determine the type of activation state*.

### Effect of disease and trauma on macrophage activation

Blood monocytes from healthy volunteers do not usually need to produce NOS or arginase, so it is not surprising that many studies have not detected NOS or arginase in these cells (10, 14, 21, 22, 29, 30, 37). However, studies performed on tissue or cells from people undergoing stress, trauma [e.g., burns (29)], pregnancy (16), or disease {such as infection [e.g., tuberculosis (12, 24, 26) or filarial infection (28)], atherosclerosis (7, 10, 17, 25), autoimmune diseases (27, 36) and cancer (42, 61)} demonstrate that human macrophages (and sometimes monocytes) can produce active forms of the arginine-metabolizing enzymes (Table 1).

Trauma results in a pattern of gene expression in macrophages that is consistent with a wound-healing response, with an initial increase in NOS followed by decreased NOS production and activity, elevated IL-4, IL-10, and TGFβ levels, and increased arginase expression and activity, resulting in decreased plasma arginine levels (28, 29, 62).

Disease, however, causes different patterns of gene expression. For example, monocytes from multiple sclerosis sufferers not only have higher levels of arginase-1 and increased urea production, but also have increased NOS2 mRNA and nitrite production (particularly when stimulated by M1 cytokines or LPS) (36). Macrophages from patients with inflammatory diseases, such as tuberculosis, malaria, or rheumatoid arthritis, have increased levels of NOS2 mRNA and active protein (4, 8, 24, 26, 63), which may contribute to elevated plasma NO levels (64). Atherosclerosis is another inflammatory disease with a considerable macrophage contribution, with oxidized low-density lipoproteins taken up by macrophages during their transformation into foam cells. Plaque macrophages express NOS2 RNA and protein, as well as markers of NOS activity (including the presence of nitrotyrosine or ceroid) (4, 7, 17, 25). Plaque macrophages and foam cells express arginase-1 (10), and macrophages laser-dissected from plaque have upregulated levels of arginase-2 and NOS2 (65). Macrophages present in some neoplastic diseases also produce active NOS2 (4, 42, 66). Reducing the local levels of arginine has been proposed as a treatment for these diseases, by reducing inflammation-triggered immune dysfunction, tumor escape, fibrosis, and immunosuppression (61). Possible pharmacological interventions include treatment with arginine degrading enzymes, NOS competitors and inhibitors, asymmetric dimethylarginine, NO-releasing aspirins, cyclooxygenase, and phosphodiesterase or arginase inhibitors (8, 61). *These studies suggest that an inflammatory environment is necessary in order to observe NOS or arginase in human monocytes and macrophages. The in vitro experiments that do not demonstrate arginase or NOS expression may simply be lacking the additional cues needed for expression rather than demonstrating an inability to actually express these factors*.

## Conclusion

The modulation of macrophages to express NOS or arginase has clear benefits for treating disease in humans (and other species). To do this, one needs to either determine suitable signals to stimulate these pathways or obtain a sufficient number of human macrophages (e.g., by tissue culture) that function like tissue macrophages.

Because macrophages from different inbred strains of mice vary greatly in their macrophage NOS and arginase balance, one would predict similar variability to be found in humans as well. In addition, the source of the macrophages being studied has been found to be important. Several groups have reported that human monocytes from healthy volunteers that have been differentiated or manipulated *in vitro* using current protocols tend not to have detectable levels of arginase and NOS enzymes, whereas MDM from diseased or stressed individuals or tissue macrophages obtained from normal, diseased, or stressed individuals do express NOS and/or arginase. Together these observations suggest that the current system of differentiating macrophages from human peripheral monocytes *in vitro* needs further refinement before it can be considered to be an accurate model of human macrophage behavior *in vivo* (63). In turn, we need to understand the differences and similarities between the different species and the cells being studied to develop experimental models that will answer some of the outstanding questions regarding macrophage M1/M2 or other activation states: What regulates macrophage activation in tissues? What mechanisms regulate macrophage plasticity and stability? How does plasticity of phenotype affect tissue macrophages? What are the full *in vivo* ramifications of the M1/M2 paradigm?

Further work is important to be sure that our observations of the human system *in vitro* are real, and not due to our cell source, measurements, or manipulations. We suggest that macrophages obtained from mice remain useful for investigating aspects of these questions in humans/human macrophages. So, although mice are not men (as Robert Koch observed), we agree with Rudolf Virchow that *“*Between animal and human medicine there is no dividing line – nor should there be. The object is different but the experience obtained constitutes the basis of all medicine*”* [Rudolph Virchow, 1821–1902].

## Conflict of Interest Statement

The authors declare that the research was conducted in the absence of any commercial or financial relationships that could be construed as a potential conflict of interest.
